# Polysaccharide extract of *Hedysarum alpinum* exhibits neuroprotective effects in an Alzheimer's disease model mice and suppresses inflammation in LPS‐induced RAW264.7 macrophages

**DOI:** 10.1002/alz70855_101570

**Published:** 2025-12-23

**Authors:** Chun‐Yen Yang, Aldarmaa Jalsrai, Hsiu‐Mei Hsieh

**Affiliations:** ^1^ National Taiwan Normal University, Taipei, Taipei, Taiwan; ^2^ Institute of Traditional Medicine and Technology, Ulaanbaata, Mongolia; ^3^ National Taiwan Normal University, Taipei, Taiwan

## Abstract

**Background:**

Alzheimer's disease (AD), the most common neurodegenerative disorder, is characterized by a progressive decline in cognitive functions. Key pathological features of AD include the accumulation of Aβ plaques, the formation of neurofibrillary tangles (NFTs) composed of hyperphosphorylated Tau, and significant neuronal loss. Neuroinflammation is critical to the pathogenesis of AD, closely linked to the development of these pathological hallmarks and the progression of neuronal damage. *Hedysarum*, a traditional herbal medicine and dietary supplement with a long history of clinical application, is widely recognized for its health‐promoting and disease‐managing properties.

**Methods:**

In this study, we investigated the neuroprotective and anti‐inflammatory potential of *Hedysarum alpinum* L. polysaccharide extracts (HAP) using two models, in vivo 5xFAD mice and in vitro LPS‐induced RAW264.7 macrophages. The 4‐month‐old 5xFAD and wild‐type (C57BL/6J) mice were orally administered with HAP (20 mg/kg) or saline vehicle daily for six weeks, respectively. Behavioral tests were conducted during the last two‐week treatment to evaluate cognitive functions.

**Results:**

In the Barnes maze, the TG+HAP group exhibited a significantly shorter latency to locate the escape hole during the training phase compared to the TG+saline group. During the probe phase, the HAP‐treated group also spent considerably more time in the target quadrant, indicating improved spatial learning and memory. Moreover, in the Y‐maze test, 5xFAD mice showed a significantly reduced spontaneous alternation rate compared to wild‐type mice, reflecting impaired short‐term memory. However, HAP administration significantly improved the spontaneous alternation rate in 5xFAD mice. Furthermore, HAP effectively attenuated LPS‐induced activation of the NLRP3 inflammasome in RAW264.7 macrophages by inhibiting the NFκB signaling pathway. HAP significantly reduced LDH release and gene expression levels of pro‐inflammatory mediators, including IL‐1β, IL‐6, TNF‐α, and iNOS.

**Conclusions:**

Our findings demonstrate that HAP significantly reduces inflammation and improves cognitive function in 5xFAD mice. These beneficial effects could be mediated through the modulation of the microbiota‐gut‐brain axis, which will be further elucidate through analyzing of the gut microbiome composition.

